# Google Trends in Dermatology: Scoping Review of the Literature

**DOI:** 10.2196/27712

**Published:** 2021-05-25

**Authors:** Torunn Elise Sivesind, Mindy D Szeto, William Kim, Robert Paul Dellavalle

**Affiliations:** 1 Department of Dermatology University of Colorado School of Medicine Aurora, CO United States; 2 University of Colorado School of Medicine Aurora, CO United States; 3 Dermatology Service Rocky Mountain Regional VA Medical Center Aurora, CO United States

**Keywords:** Google Trends, search trends, internet, infodemiology, infoveillance, search terms, dermatology, skin cancer, databases

## Abstract

**Background:**

Google Trends is a powerful online database and analytics tool of popular Google search queries over time and has the potential to inform medical practice and priorities.

**Objective:**

This review aimed to survey Google Trends literature in dermatology and elucidate its current roles and relationships with the field.

**Methods:**

A literature search was performed using PubMed to access and review relevant dermatology-related Google Trends studies published within the last 5 years.

**Results:**

Current research utilizing Google Trends data provides insight related to skin cancer, pruritus, cosmetic procedures, and COVID-19. We also found that dermatology is presently the highest-searched medical specialty—among 15 medical and surgical specialties as well as general practitioners. Google searches related to dermatology demonstrate a seasonal nature for various skin conditions and sun-related topics, depending on a region’s inherent climate and hemi-sphere. In addition, celebrity social media and other viral posts have been found to potentiate Google searches about dermatology and drive public interest.

**Conclusions:**

A limited number of relevant studies may have been omitted by the simplified search strategy of this study, as well as by restriction to English language articles and articles indexed in the PubMed database. This could be expanded upon in a secondary systematic review. Future re-search is warranted to better understand how Google Trends can be utilized to improve the quality of clinic visits, drive public health campaigns, and detect disease clusters in real time.

## Introduction

Google Trends is an online database and analytics tool provided by Google (Google LLC, Mountain View, CA) that reports the relative popularity of a particular search term on Google compared to the peak number of Google searches related to that term over a certain period of time [1,2]. A search volume index (SVI) between 0 to 100 is then generated [2,3], reflecting trends and peak popularity for the term over a given timespan. Data are normalized by Google Trends such that ratios are created, which relate the search volume of an individual search term to the aggregate search volume for all queries, for a given geographic location and time range. These ratios are then proportionally transformed to a scale of 0 to 100 based on a search term’s relative magnitude compared to all searches [2,4].

It is also possible to find the true search volume (rather than relative volume) by using the Google Keyword Planner [4], which allows one to assess a particular term for its total number of Google searches over a specified timeframe in a specific location. Patterns and changes in topic popularity may be examined via investigating different time periods or different locations after separately downloading each result and then comparing these side-by-side. In a similar manner, it is also possible to compare the relative popularity of topics by conducting separate searches utilizing different search terms.

Recently, many medical specialties, including dermatology, have started using Google Trends data to better understand population interest in various topics, such as sunscreen, sunburn, skin cancer, and melanoma [1]. Knowledge gained from Google Trends has the potential to guide public health interventions, improve patient education, and tailor health care to patient concerns. Google Trends data are of particular interest to the field of dermatology; in a 2018 study, Ransohoff and Sarin [5] found that, among medical specialists and general practitioners, the most frequently searched physicians were dermatologists.

This study was undertaken to provide a scoping [6] preliminary review of literature in the field of dermatology that highlights the use of Google Trends, to elucidate the current relationship between dermatology and Google Trends, describe limitations of its use, and guide future directions for research and application to patient care.

## Methods

A literature review was performed using the PubMed database to tabulate the total number of Google Trends studies from 2016 to 2020. To isolate the relevant dermatology-related publications, the following search string was utilized on August 4, 2020, resulting in 53 articles for initial screening: ((((“Dermatology”[Mesh]) OR “Skin Diseases”[Mesh]) OR “Cosmetic Techniques”[Mesh]) OR “Cosmeceuticals”[Mesh]) AND (google trends AND (y_5[Filter])). The search term “y_5” was included to filter articles published within the last 5 years (2016-2020). Screening of title and abstract for these articles was performed independently by 2 authors with education and experience in dermatology (TS, WK), and of these, 26 were ultimately chosen for inclusion in this review after determination of relevance.

## Results

The total number of Google Trends publications over the last 5 years, along with the dermatology-related subset of 26 articles screened is displayed and tabulated by year in Multimedia Appendix 1.

Summary information for the 26 dermatology-specific articles included in this review is captured in Table 1**.**

**Table 1:** Summary of dermatology-related Google Trends publications (2016-2020) included in the review.

Topic and article citation			Google Trends location(s) analyzed		Key findings
**Skin-related topics of public interest**					
	Ransohoff JD, Sarin KY. Referred by Google: mining Google Trends data to identify patterns in and correlates to searches for dermatological concerns and providers. Br J Dermatol; 2018 [5]	US, UK, South Africa, Singapore, worldwide		During the study window (September 2011-September 2016), searches originating in the United States revealed that dermatology is the most frequently searched medical specialty, followed by psychiatry and ophthalmology. This correlates with the proportion of self-referred patients, not with overall volume of visits. Countries in the southern hemisphere, such as South Africa, demonstrate a reversed seasonal search trend for dermatologists compared to countries in the northern hemisphere. Countries without distinct seasons, such as Singapore, had no seasonal variation in searches.	
	Hopkins ZH, Secrest AM. An international comparison of Google searches for sunscreen, sunburn, skin cancer, and melanoma: Current trends and public health implications. Photodermatol Photoimmunol Photomed; 2019 [1]	US, UK, Canada, Australia, New Zealand		From January 1, 2004 to December 31, 2017, sunscreen and sunburn had the highest intraterm correlation, with both searches rising. Searches for skin cancer and melanoma decreased during the study window for all countries except New Zealand and the United Kingdom. Countries with rising rates of melanoma displayed higher searches for all terms. Recommended online skin cancer prevention campaigns use the search terms “sunburn” and “sunscreen.”	
	Seidl S, Schuster B, Rüth M, Biedermann T, Zink A. What do Germans want to know about skin cancer? A nationwide Google search analysis from 2013 to 2017. J Med Internet Res; 2018 [7]	Germany		From November 2013 to October 2017, the highest volume search terms related to nonmelanoma skin cancer and melanoma included “skin cancer,” “white skin cancer,” “basalioma,” and “melanoma.” The most-searched anatomic area of nonmelanoma skin cancer was ““nose” and for melanoma, “nails.” This suggested that this information can be utilized to focus educational campaigns.	
	Callaghan DJ. Use of Google Trends to examine interest in Mohs micrographic surgery: 2004 to 2016. Dermatol Surg; 2018 [8]	US		During the study window (2004-2016), there was a rise in searches for “Mohs surgery,” positively correlated with a rise in the SVI^a^ for “basal cell carcinoma” and “squamous cell carcinoma,” but without a similar correlation for “melanoma” or “skin cancer.”	
	Huang X, Baade P, Youlden DR, Youl PH, Hu W, Kimlin MG. Google as a cancer control tool in Queensland. BMC Cancer; 2017 [9]	Australia		Increases in the monthly ASR^b^ for breast cancer, melanoma, and prostate cancer were significantly correlated with increases in monthly SVIs. Colorectal cancer did not show this significant correlation. However, the predictive powder of SVIs to account for ASR variances was different for each cancer type, suggesting that more research is needed for internet search–based cancer surveillance.	
	Zink A, Schuster B, Rüth M, Pereira MP, Philipp-Dormston WG, Biedermann T, Ständer S. Medical needs and major complaints related to pruritus in Germany: a 4-year retrospective analysis using Google AdWords Keyword Planner. J Eur Acad Dermatol Venereol; 2019 [10]	Germany		During the study window (June 2013-April 2017), the most searched German language Google terms on the topic of pruritus included “atopic eczema,” the lay term for psoriasis; “Schuppenflechte”; and “psoriasis.” A seasonal trend was noted, with the lowest searches for pruritus occurring in the summer.	
	Motosko C, Zakhem G, Ho R, Saadeh P, Hazen A. Using Google to trend patient interest in botulinum toxin and hyaluronic acid fillers. J Drugs Dermatol; 2018 [11]	US		Search volume for queries related to neuromodulators and hyaluronic acid–based fillers was positively correlated with the number of procedures performed in the same year and year prior.	
	Wang S, Manudhane A, Ezaldein HH, Scott JF. A review of the FDA's 510(k) approvals process for electromagnetic devices used in body contouring. J Dermatolog Treat; 2019 [12]	US		Google Trends SVI for the term “Emsculpt” grew during 2018, reaching a peak in July.	
	Seth D, Wang S, Ezaldein HH, Merati M, Scott JF. Over-the-counter light therapy for acne: a cross-sectional retrospective analysis. Dermatol Online J; 2019 [13]	US		During the study window (2010-2018), SVI for “acne light therapy mask” peaked in 2016.	
	Dey M, Zhao SS. COVID-19 and Kawasaki disease: an analysis using Google Trends. Clin Rheumatol; 2020 [14]	Worldwide		Searches for “Kawasaki disease” rose sharply in April 2020; “Kawasaki disease” SVI was highest in Europe, possibly reflecting pediatric manifestation of COVID-19 in European populations. This suggests the use of Google Trends to detect disease clusters in real time.	
**Seasonality of searches**					
	Kardeş S. Seasonal variation in the internet searches for psoriasis. Arch Dermatol Res; 2019 [15]	US, UK, Canada, Ireland, Australia, New Zealand		Public interest in psoriasis-related information shows seasonal variability, with interest peaking in late winter and early spring.	
	Zink A, Schuster B, Rüth M, Pereira MP, Philipp-Dormston WG, Biedermann T, Ständer S. Medical needs and major complaints related to pruritus in Germany: a 4-year retrospective analysis using Google AdWords Keyword Planner. J Eur Acad Dermatol Venereol; 2019 [10]	Germany		During the study window (June 2013-April 2017), the most searched German language Google terms on the topic of pruritus included “atopic eczema,” the lay term for psoriasis” “Schuppenflechte”; and “psoriasis.” A seasonal trend was noted, with the lowest searches for pruritus occurring in the summer.	
	Tizek L, Schielein M, Rüth M, Ständer S, Pereira MP, Eberlein B, Biedermann T, Zink A. Influence of Climate on Google Internet Searches for Pruritus Across 16 German Cities: Retrospective Analysis. J Med Internet Res; 2019 [16]	Germany		Between August 2014 and July 2018, “pruritis” and “anal pruritis” were the most searched terms for the topic of pruritis, with more searches related to chronic than to acute pruritis. Temperature had a larger effect on searches than particle matter, humidity, and sunshine duration, with a peak in searches during winter.	
	Kirchberger MC, Kirchberger LF, Eigentler TK, Reinhard R, Berking C, Schuler G, Heinzerling L, Heppt MV. Interest in tanning beds and sunscreen in German-speaking countries. J Dtsch Dermatol Ges; 2017 [17]	Germany, Austria, Switzerland		During the study window (2004-2016), worldwide searches for sunscreen increased, while tanning bed searches decreased. Conversely, for German-speaking countries, there were more searches for tanning beds than for sunscreen. More education regarding prevention of UV^c^ damage is needed for those residing in German-speaking countries.	
	Toosi B, Kalia S. Seasonal and geographic patterns in tanning using real-time data from Google Trends. JAMA Dermatol; 2016 [18]	Canada, US, Australia		Public interest in tanning salons and tanning beds has been declining since 2010 in Canada and since 2012 in the United States and Australia. Interest in tanning is seasonal — highest in March in the northern hemisphere, highest in September in the southern hemisphere.	
	Ransohoff JD, Sarin KY. Referred by Google: mining Google Trends data to identify patterns in and correlates to searches for dermatological concerns and providers. Br J Dermatol; 2018 [5]	US, UK, South Africa, Singapore, worldwide		During the study window (September 2011-September 2016), searches originating in the United States revealed that dermatology is the most frequently searched medical specialty, followed by psychiatry and ophthalmology. This correlates with the proportion of self-referred patients, not with overall volume of visits. Countries in the southern hemisphere, such as South Africa, demonstrate a reversed seasonal search trend for dermatologists compared to countries in the northern hemisphere. Countries without distinct seasons, such as Singapore, were found to have no seasonal variation in searches.	
	Kluger N. Insights into worldwide interest in tattoos using Google Trends. Dermatology; 2019 [19]	Worldwide		Public interest in tattoos increased steadily during the study window (January 2004-December 2018). Interest in tattoos demonstrates a seasonal pattern, peaking in the summer months.	
	Celaj S, Deng J, Murphy BL, Kundu RV. Analysis of population inquiry on practices for ultraviolet radiation protection. Dermatol Online J; 2017 [20]	US		During the study window (2004-2015), “broad spectrum sunscreen” SVI was highest in June and lowest in winter. After an FDA^d^ announcement in 2011 regarding broad spectrum sunscreen labeling, the SVI for “broad spectrum sunscreen” increased. The “sunblock” and “sunscreen” SVIs were highest in June and lowest in December but were not affected by the FDA 2011 announcement. This suggests that Google Trends can be utilized to monitor campaigns or public health policy changes similar to the 2011 FDA announcement.	
	Kluger N, Bouchard LJ. A comparative study of Google Search trends for melanoma, breast cancer and prostate cancer in Finland. Dermatology; 2019 [21]	Finland-based; included data from Belgium, Italy, Portugal, and Sweden		Compared public interest in melanoma to breast and prostate cancer, using Google Trends (January 1, 2010-January 1, 2019). The “melanoma” SVI was maximal in early summer. SVI peaks for the terms related to breast and prostate cancer were positively correlated with awareness campaigns for those diseases. This suggests a second public health campaign for melanoma awareness should be timed during winter (lowest public interest period).	
	Xu S, Thyssen JP, Paller AS, Silverberg JI. Eczema, atopic dermatitis, or atopic eczema: analysis of global search engine trends. Dermatitis; 2017 [22]	English, Russian, Japanese, Turkish, German (specified languages searched)		From 2014 to 2016, searches for eczema increased, while “atopic dermatitis” and “atopic eczema” searches remained stable. Authors recommended the universal use of “atopic dermatitis” rather than “eczema” due to the term’s ambiguous nature. Seasonal climate changes were associated with flares of severity for atopic dermatitis and corresponded with search trends.	
	Hsiang EY, Semenov YR, Aguh C, Kwatra SG. Seasonality of hair loss: a time series analysis of Google Trends data 2004-2016. Br J Dermatol; 2018 [3]	8 English-speaking countries (did not specify)		SVI for terms related to hair loss demonstrated seasonality, with peaks in summer and autumn. This suggests symptoms of hair loss are greatest in summer and autumn.	
**Social media's impact on searches**					
	Ward B, Ward M, Paskhover B. Google Trends as a resource for informing plastic surgery marketing decisions. Aesthetic Plast Surg; 2018 [23]	US		Highly publicized plastic surgery events and celebrity social media posts have a significant impact on search volume for related topics.	
	Noar SM, Leas E, Althouse BM, Dredze M, Kelley D, Ayers JW. Can a selfie promote public engagement with skin cancer? Prev Med; 2018 [24]	US		Searches related to skin cancer, skin cancer prevention, and tanning increased during the period May 13-17, 2005, after a cancer selfie Facebook post went viral. This suggests that public health practitioners can utilize Google Trends to build on viral posts in real time to potentiate positive health benefits.	
**Limitations and strengths of Google Trends**					
	Martinez-Lopez A, Ruiz-Villaverde R, Molina-Leyva A. Google search trends in psoriasis: a pilot evaluation of global population interests. J Eur Acad Dermatol Venereol; 2018 [25]	Worldwide		This was a pilot study to assess primary topics of interest to those with psoriasis. The search term with the most growth during the period 2004-2016 was “kim kardashian psoriasis.”	
	Kluger N. Tattoo side effects worldwide: a Google Trends-based time series analysis. Acta Dermatovenerol Alp Pannonica Adriat; 2019 [26]	Worldwide		SVI for tattoo-related symptoms increased during the study window (January 1, 2004-December 31, 2018).	
	Corazza M, Amendolagine G, Musmeci D, Forconi R, Borghi A. Sometimes even Dr Google is wrong: An unusual contact dermatitis caused by benzoyl peroxide. Contact Dermatitis; 2018 [27]	Italy		The study compared SVI for 3 chronic diseases to searches for “dermatitis,” noting that only “diabetes” was searched more frequently than “dermatitis.” This demonstrates likelihood of self-diagnosis of dermatitis via the internet. Search volume for the medication Benzac peaked in autumn and winter, aligning with seasonal acne flares.	
	Hessam S, Salem J, Bechara FG, Haferkamp A, Heidenreich A, Paffenholz P, Sand M, Tsaur I, Borgmann H. Hidradenitis suppurativa gains increasing interest on World Wide Web: a source for patient information? Int J Dermatol; 2017 [28]	Worldwide		Search queries related to HS^e^ have risen over the last decade. HS-related website analysis demonstrates a need for improvement in quality and readability to raise disease awareness and allow earlier patient presentation for undiagnosed patients.	
	Lospinoso DJ, Lospinoso JA, Miletta NR. The impact of ultraviolet radiation on sunburn-related search activity. Dermatol Online J; 2017 [29]	US		A strong positive association between the UV index and SVI for sunburn-related terms was demonstrated. This suggests tracking search terms for sun protective measures as an indication of public awareness and efficacy of public health programs.	

^a^SVI: search volume index.

^b^ASR: age-standardized incidence rate.

^c^UV: ultraviolet.

^d^FDA: Food and Drug Administration.

^e^HS: hidradenitis suppurativa.

## Discussion

### Skin-Related Topics of Public Interest

Notably, dermatology was reported by Ransohoff and Sarin [5] to be the most searched medical specialty in a comparative study of 15 medical specialties. To compare relative specialist popularity using Google Trends data, the study [5] used the search term “physician,” to enable all specialist search terms to be normalized to the SVI of this term (the most popular term) and thus enabled an accurate comparison across all 15 physician specialties. However, the study by Ransohoff and Sarin [5] also found that, despite being the most searched specialty, dermatology ranked sixth in number of clinic visits per specialty and revealed that dermatologists had one of the highest percentages of self-referred patients. The authors concluded that the high rate of dermatology Google searches correlates with the proportion of self-referred patients rather than the number of clinic visits, and therefore, changes in online search volume may better reflect how patients initiate care with dermatologists rather than demonstrate real-time vacillations in clinic volume [5].

Several papers also describe the use of Google Trends to gather information about user searches related to skin cancer and skin cancer prevention. Google Trends searches for “sunscreen” and “sunburn” are growing in the United States, United Kingdom, Canada, Australia, and New Zealand [1]. Conversely, searches for “skin cancer” and “melanoma” are declining for one or both terms in the United States, Canada, and Australia [1].

In Germany, the most searched term in the skin cancer–related category was “forms of skin cancer,” followed by “skin alterations” [7]. Seidl et al [7] also noted that the most searched anatomic locations of melanoma were “nails” and “eyes,” while for nonmelanoma skin cancer, the most searched locations were “nose” and “face.” A 2018 study [8] of US-based Google searches discovered that the search terms “Mohs surgery,” “basal cell carcinoma,” and “squamous cell carcinoma” rose during the years 2004 to 2016. This finding aligns with a rise in the use of Mohs micrographic surgery during this time, according to reports from Medicare and the National Ambulatory Medical Survey [8].

A study by Huang et al [9] using data from the Queensland Cancer Registry from January 2006 to December 2012 reported that increases in the monthly age-standardized incidence rates (ASR) for melanoma correlated with increases in the monthly SVI for search terms “skin cancer” and “melanoma.” Additionally, through use of a multiple linear regression model, Huang et al [9] reported that the predictive power of the “skin cancer” SVI to explain variance in melanoma ASR was 17.9%, noting that the search term “skin cancer” includes both melanoma and keratinocyte carcinomas and thus may better predict rates of melanoma than the specific search term “melanoma,” since online users may not be able to distinguish different skin cancer types when performing Google searches.

Google Trends has also been utilized to study pruritus. According to a 2019 study conducted in Germany by Zink et al [10] that utilized the Google Keywords Planner to determine search volume, it was discovered that for German language Google searches regarding pruritus, the most searched terms included “atopic eczema” (24.3%), the common term for psoriasis, “Schuppenflechte” (17.8%), “psoriasis” (13.4%), and the German word for pruritus, “Juckreiz” (2.9%).

Gauging public interest in certain cosmetic procedures and treatments is another application of Google Trends data. A 2018 study by Motosko et al [11] found that the number of procedures employing botulinum toxin and/or hyaluronic acid–derived fillers correlated significantly with the search volume for related search terms over the period spanning 2005 to 2016, while Wang et al [12] noted search volume expansion for “Emsculpt,” a body contouring technique, during 2018. Similarly, a study by Seth et al [13] reported an increase in the search term “acne light therapy mask” in 2016, reflecting a parallel increase in public interest in light-emitting diode therapy for inflammatory acne.

Of particular interest given the currently evolving pandemic, a 2020 study by Dey and Zhao [14] examined the relationship between COVID-19 and Kawasaki disease using Google Trends data. The study found that the search frequencies of Kawasaki disease in 2019 and 2020 were similar until April 2020, when reports of a Kawasaki-like disease in children with COVID-19 emerged. From February to May 2019, searches for Kawasaki disease were highest in Indonesia, the Philippines, and Malaysia — regions where the disease is more commonly found. However, from February to May 2020, searches for Kawasaki disease were highest in France, Switzerland, and Italy, a finding that reflects our current knowledge of the multisystem inflammatory syndrome in children (MIS-C), associated with COVID-19 and aligning with MIS-C clusters in Europe during this time [22,30]. While popularity of search terms may be confounded by news or social media–driven popularity, Dey and Zhao [14] noted that the association revealed through Google Trends data could highlight its potential future utility in identifying real-time disease clusters.

### Seasonality of Searches

Google searches related to dermatology demonstrate a seasonal nature for several skin diseases. A 2019 study by Kardeş [15] found that searches for “psoriasis” were elevated in the late winter and early spring and were lower in the late summer and early fall, with statistically significant results from the United Kingdom, Canada, Ireland, Australia, and New Zealand and noted a trend toward seasonal variation in US-based searches.

In Germany, Google searches for “pruritus” were lowest in the summer [10] and highest in the winter [16]. Additionally, “tanning bed” was searched most in March and May, while “sunscreen” search volume was highest in June [17]. Toosi and Kalia [18] reported that public interest in tanning for Canada and the United States was highest in March and for Australia, was highest in September. Countries in the southern hemisphere, such as South Africa, demonstrate a reversed seasonal search trend compared to countries in the northern hemisphere; countries without distinct seasons, such as Singapore, were found to have no seasonal variation in searches [5]. According to Kluger [19], the term “tattoo” was searched most during the summer and least during winter: Searches for the term “tattoo” are highest from July to August in the northern hemisphere and reach a peak in January for regions in the southern hemisphere. Interestingly, Kluger [26] found that searches for symptoms of tattoo-related complications, such as pruritus and induration, rose during the period 2004 through 2018 inclusive — however, interpretation of these results is difficult, as the search volume increase could reflect either a real increase in tattoo side effects or be secondary to rising popularity of tattoos [26].

Search patterns for sunscreen in the United States have shown similar seasonal trends [20]. A US-based study found the search terms “skin cancer” and “tanning” were highest in the early summer months, while “sunburn” was highest in the late summer; the monthly timing of these trends was noted to be reversed in countries of the southern hemisphere, such as South Africa [5]. “Melanoma” was found to have the highest searches in the early summer months of either hemisphere [5,21].

In a study of worldwide Google Trends data spanning 5 languages, 4 of these demonstrated peaks in SVI for the term “eczema” in line with seasonal climate changes that are associated with flares of severity for atopic dermatitis, speaking to both the seasonality of searches and to the widespread usage of “eczema” as a synonym for atopic dermatitis [31]. Hsiang et al [3] studied “hair loss” search volume, with data from the top 4 most populous countries of both hemispheres and observed the highest SVI occurred in the summer and autumn, with the lowest search volume corresponding to the spring. The authors noted these results suggest a correlative relationship between hair loss and seasonality and that hair loss is greatest in the summer and autumn. This is in line with other studies that have noted the percentage of telogen phase hairs peaks in the late summer, with a smaller peak in the spring, and therefore, maximal hair loss occurs at the end of summer and during the fall [32].

### Social Media’s Impact on Searches

Social media has been found to influence dermatology-related search volume on Google. A 2018 study by Ward et al [23] found that the average interest level of fillers increased by 30.31 points (relative to a maximum SVI of 100) in the time period after Kylie Jenner stated that she underwent lip augmentation with Juvederm in April 2015, compared to searches conducted in the period prior (January 2004 through March 2015). Additionally, a study by Noar et al [24] noted that after a highly graphic skin cancer selfie went viral on Facebook, all search queries for skin cancer, skin cancer prevention, and tanning significantly increased, with May 13, 14, and 15 of 2017 being the 6th, 8th, and 40th most searched days for skin cancer since the inception of Google Trends on January 1, 2004.

### Limitations and Strengths of Google Trends

The main limitation of utilizing Google Trends to accurately assess public interest in a certain dermatologic topic is that it relies on the ability to access the internet, which is variable [25,26]. Individuals who are of lower socioeconomic status and educational background may not have access to a computer or smart device through which they can use Google. Additionally, individuals who are of older age may choose not to use a computer and thus would not use Google. As a result, certain groups of individuals who may be at higher risk for particular dermatologic conditions may not use Google and are omitted from Google Trends. This would imply that the dermatologic interests of people in older age groups or lower socioeconomic status may not be accurately or fully represented in Google Trends data, which could bias the information it presents and limits extrapolation to the broader population.

Thus, while Google Trends is a powerful tool for assessing the public’s dermatologic interest, it is important to consider which populations are unequally represented with this instrument and find other tools to fill these gaps. While Google accounts for approximately 91% of the market share of online searches worldwide (according to available data from January 2021), it is vital to note that search engines other than Google may be utilized, along with specific health information websites; therefore, analysis of a variety of search engines could potentially capture a more complete picture of current trends in skin-related topics of public interest.

Nevertheless, Google Trends may be a useful tool for dermatologists to gauge areas of skin concern and topics of interest, as patients may perform searches on Google for questions they are not comfortable asking their dermatologists in a clinic visit [11]. Additionally, the increasing use of Google to search skin diseases may highlight the increased prevalence of self-diagnosis and self-treatment [27,28]. Therefore, it could be useful for professional medical organizations to take note of which skin diseases and dermatology-related terms are most often searched and to notify individual dermatologists, who could then dedicate additional time to patient education, reaffirm facts, and dispel misconceptions and misinformation.

One potential public health benefit of Google Trends would be to focus on highly searched terms in order to guide and drive health campaigns. For example, in 2019, Hopkins and Secrest [1] recommended online skin cancer prevention campaigns to focus on the search terms “sunburn” and “sunscreen” as these 2 terms have demonstrated increasing search frequency from 2004 to 2016. These public health campaigns can be specifically tailored to complement and make the most of current public interest [7]. Frequency of searched terms can serve as a proxy for public awareness and speak to the efficacy of public health programs [29].

Another possible use of Google Trends is for dermatologists to quickly detect the presence of viral social media content related to dermatology, such as the skin cancer selfie that went viral on Facebook in 2017, and to then engage with the online public and utilize this viral content to strengthen public health campaigns [24]. Lastly, as the study by Dey and Zhao [14] of COVID-10 and Kawasaki-like disease illustrates, Google Trends has the potential to be utilized in identifying disease clusters in real time, meriting further research to better understand how dermatology may leverage this tool in early identification of public health concerns in the future.

### Limitations of This Scoping Review

Limitations of this review article include its simplified search strategy, which included only English language publications, restriction to the PubMed database, and lack of previous studies examining Google Trends and dermatology. Google Trends is a relatively new tool (2006 conception), and therefore, not many dermatology publications highlight the use of Google Trends in dermatology. For example, in 2020, there was only 1 dermatology Google Trends publication out of 174 total publications discussing Google Trends, as illustrated in Multimedia Appendix 1. This highlights the gap in prior literature on the intersection of these 2 topics, which weakens the conclusions we can generate from existing research. While we lay the groundwork with this survey and scoping review of related literature [6], in order to better assess the utility of Google Trends in dermatology, further research in this area is required.

### Summary

Review of the literature illustrates that assessment of Google Trends data related to dermatology has been studied, with topics including skin cancer, pruritus, cosmetic procedures, social media, clinic visits, and the associated Kawasaki-like disease of COVID-19 (MIS-C). Additionally, a seasonal nature of search terms has been widely reported in the literature, varying according to hemisphere. The main limitation of Google Trends as a research tool is access to and use of the internet. Further research is needed to better understand how this search tool can be utilized to improve the quality of clinic visits, drive public health campaigns, and detect disease clusters in real time.

## Appendix

Multimedia Appendix 1 Number of dermatology-related publications compared to the total number of Google Trends publications by year (2016-2020).

## Abbreviations

ASR: age-standardized incidence rate
MIS-C: multisystem inflammatory syndrome in children
SVI: search volume index
