# Supplementary figures and images for: Google Trends in Dermatology: Scoping Review of the Literature
Source: JMIR Dermatol. 2021 May 25;4(1):e27712. doi: 10.2196/27712 (PMC10501516; doi:10.2196/27712)

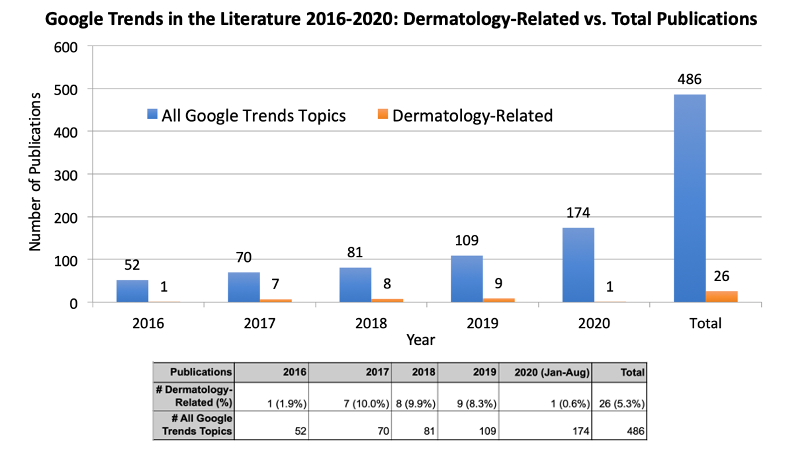

Supplement: Multimedia Appendix 1 [file derma_v4i1e27712_app1.png]
